# Protein encoded by oncogene *6b* from *Agrobacterium tumefaciens* has a reprogramming potential and histone chaperone-like activity

**DOI:** 10.3389/fpls.2014.00572

**Published:** 2014-10-28

**Authors:** Nanako Ishibashi, Saeko Kitakura, Shinji Terakura, Chiyoko Machida, Yasunori Machida

**Affiliations:** ^1^Division of Biological Science, Graduate School of Science, Nagoya UniversityNagoya, Japan; ^2^Graduate School of Bioscience and Biotechnology, Chubu UniversityKasugai, Japan

**Keywords:** tumorigenesis, oncogene *6b*, meristematic states, cell division potential, reprogramming of plant cells

## Abstract

Crown gall tumors are formed mainly by actions of a group of genes in the T-DNA that is transferred from *Agrobacterium tumefaciens* and integrated into the nuclear DNA of host plants. These genes encode enzymes for biosynthesis of auxin and cytokinin in plant cells. Gene *6b* in the T-DNA affects tumor morphology and this gene alone is able to induce small tumors on certain plant species. In addition, unorganized calli are induced from leaf disks of tobacco that are incubated on phytohormone-free media; shooty teratomas, and morphologically abnormal plants, which might be due to enhanced competence of cell division and meristematic states, are regenerated from the calli. Thus, the *6b* gene appears to stimulate a reprogramming process in plants. To uncover mechanisms behind this process, various approaches including the yeast-two-hybrid system have been exploited and histone H3 was identified as one of the proteins that interact with 6b. It has been also demonstrated that 6b acts as a histone H3 chaperon* in vitro* and affects the expression of various genes related to cell division competence and the maintenance of meristematic states. We discuss current views on a role of 6b protein in tumorigenesis and reprogramming in plants.

## INTRODUCTION

*Agrobacterium tumefaciens* strains that harbor tumor inducible (Ti) plasmids cause crown gall tumors upon infection of dicot plants. Ti plasmids of *Agrobacterium* transfer their T-DNAs to the chromosomal DNAs of plants. These T-DNAs contain three gene loci involved in this tumorigenesis, *tmr*, *tms* (*tms1* and *tms2*), and *tml*. The *tmr* and *tms* loci contain genes involved in the biosynthesis of cytokinins and auxins, respectively. The *tml* locus was originally identified as the mutated gene that causes tumors larger than those induced by the wild-type T-DNA (Garfinkel et al., 1981). It contains two genes *6a* and *6b*: the former encodes an unknown protein whereas the molecular characteristics of the latter had also remained undefined until Terakura et al. (2007) identified them recently. Gene *6b* in the *tml* loci is conserved in the T-DNA of all strains of *Agrobacterium* and is actively transcribed in the tumor cells (Willmitzer et al., 1983; Otten and De Ruffray, 1994). In addition, some plant species that were infected with *Agrobacterium* stains having T-DNAs containing only the *6b* genes showed the formation of small but significantly sized tumors at the infection sites (Hooykaas et al., 1988; Tinland et al., 1989). When *6b* was introduced into leaf disks, which were then incubated on hormone-free medium, unorganized calli were formed (Wabiko and Minemura, 1996). Transgenic plants regenerated from these calli exhibited morphological defects in various organs (Tinland et al., 1992; Wabiko and Minemura, 1996; Gális et al., 1999, 2002; Helfer et al., 2003; Terakura et al., 2006). The defects appear to be due to increased meristematic states and cell division potentials. These observations suggest that the 6b proteins play roles in the maintenance of tumorigenic states as well as the induction of dedifferentiation and differentiation (reprogramming process) of plant organs.

In the present mini review, we will summarize the results of recent studies, including our own, to present a novel understanding of the molecular characteristics of 6b protein and discuss how 6b might act as a reprogramming factor. We focus here on the subcellular localization of 6b protein; relationships between such localization and the ability of 6b to induce tumorigenic states; plant genes that are affected by 6b; plants proteins that interact with 6b; and, finally, a molecular function of 6b.

## STRUCTURAL FEATURES OF *6b* PROTEIN

**FIGURE 1:**
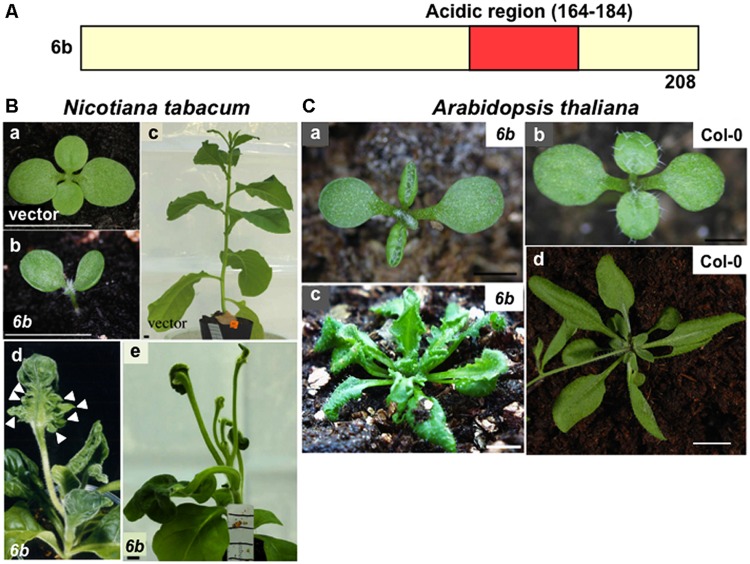
**Morphological defects in the transgenic plants that express 6b protein. (A)** Schematic domain organization of 6b protein. The acidic region (residues 164–184) is present in 6b. **(B)** Photographs showing clearly the reported phenotypes of transgenic tobacco plants. Transgenic plants transformed with the empty vector (a,c) or *6b* gene (b,d,e). The *6b*-transgenic seedling (b) and young plant (d) exhibiting mild defects and the *6b*-plant (e) exhibiting severe defects. Arrowheads indicate protrusions on the abaxial side of leaves. Scale bars: 10 mm. **(C)** Photographs showing clearly the reported phenotypes of non-transgenic and transgenic *Arabidopsis* plants. The *6b*-transgenic *Arabidopsis* plants (a,c); non-transgenic Col-0 plants (b,d). Scale bars: 1 mm (a,b), 10 mm (c,d). Some of pictures were published previously ( Terakura et al., 2006).

The *6b* gene of *A. tumefaciens* AKE10 strain (Wabiko and Minemura, 1996) encodes a protein with 208 amino acid residues that contains an acidic region (**Figure 1A**: residues 164–184) near the carboxyl terminus, suggestive of interactions with other proteins to generate complexes. The acidic region is essential for the generation of abnormal phenotypes by *6b* (Terakura et al., 2006). It is worth pointing out that when the GAL4 DNA binding domain is fused to 6b protein, the fusion protein has a transactivation activity, which requires the acidic region (Kitakura et al., 2002).

The remaining amino acid sequence of 6b exhibits weak similarity to those sequences of several proteins encoded by other genes in the Ti and Ri plasmids of *A. rhizogenes* T-DNAs, *rolB*,* rolC,* ORF13, and ORF14 that belong to the *plast* family (Spena et al., 1987; Levesque et al., 1988; Stieger et al., 2004). Despite of their weak similarities, they also induce commonly abnormal growth and morphology of both roots and shoots, and aberrant accumulation of sugars and metabolites (Cardarelli et al., 1987; Spena et al., 1987; Lemcke and Schmülling, 1998; Helfer et al., 2002; Stieger et al., 2004; Mohajjel-Shoja et al., 2011). The amino acid sequence of the ORF13 protein contains a retinoblastoma (Rb)-binding motif (LXCXE), and it binds to the maize Rb protein (Stieger et al., 2004); however, 6b does not have this motif.

Although 6b protein contains no motif similar to the nuclear localization signal (NLS), 6b protein localizes to the nucleus (Kitakura et al., 2002). Only the RolB protein in the *plast* family has also been shown to localize to plant nuclei (Moriuchi et al., 2004). Tobacco plants that express a glucocorticoid-receptor-fused 6b protein show the abnormal phenotypes described above only after being treated with dexamethasone, which stimulates nuclear import of the protein (Terakura et al., 2006). This result suggests that the nuclear localization of 6b is required for the appearance of morphological defects of plants and abnormal cell proliferation. Mechanisms of nuclear localization of the 6b protein, however, have remained unknown. There are two possible explanations for such a localization of 6b. (1) The relative molecular mass of 6b protein is sufficiently small enough (approximately 20,000) to be transported into the nucleus by passive diffusion and it might be trapped there by a certain nuclear protein. (2) As mentioned below, 6b interacts with the NtSIP1 and NtSIP2 proteins, which are tobacco transcription factor-like nuclear proteins (Kitakura et al., 2002, 2008). Therefore, 6b proteins might be transported into the nucleus through an interaction with these endogenous nuclear proteins. Regardless of the molecular mechanisms involved, however, 6b appears to play a role in the expression of some genes that might be involved in cell proliferation and differentiation.

## CHARACTERISTICS OF TRANSGENIC PLANTS THAT EXPRESS *6b*

### PHENOTYPIC CHARACTERISTICS OF TOBACCO PLANTS THAT EXPRESS *6b*

The shoots and leaves of transgenic tobacco plants derived from calli, which are induced by the expression of *6b* and cultured on hormone-free-medium, show various morphological defects (**Figure 1B**; Tinland et al., 1992; Wabiko and Minemura, 1996; Helfer et al., 2003; Terakura et al., 2006). In the tobacco transgenic plants that show mild defects, cotyledons and leaves are curled upwardly at an early growth stage (**Figure 1Bd,d**) and generate a number of outgrowths from the abaxial surface (**Figure 1Bd**). Transgenic plants that show the severe phenotype produce leaves with long petioles and an unexpanded lamina associated with rod-shaped protrusions (**Figure 1Be**). Expression of *6b* in transgenic tobacco plants also induces abnormal venation patterns of cotyledons, which sometimes include veins with inverted adaxial–abaxial polarity (Kakiuchi et al., 2007). In hypocotyles of these transgenic tobacco seedlings, basipetal auxin transport was reduced, which might be related to morphological abnormalities as described above (Kakiuchi et al., 2006). Takahashi et al. (2013) suggested that 6b protein is involved in modulating auxin and cytokinin localization and affects proliferation of tumorigenic cells expressing *6b*.

To understand the molecular basis for the upward curling of leaves and the protrusion at the leaf abaxial side of tobacco transgenic plants with the *6b* gene, Terakura et al. (2006) measured levels of *Cyclin B1* and* NACK1* transcripts (as markers for the cell cycle) in mature leaves of *6b*-transgenic plants and carried out* in situ* hybridization with sections of mature leaves and cDNAs of *NACK1*. Transcript levels of these genes were increased and these genes were ectopically transcribed on the abaxial side of mature leaves, suggesting increased cell division competence in the abaxial domain of *6b*-transgenic leaves.

In addition to aberrant morphology and patterns of cell division, expression of *6b* in tobacco plants stimulates sugar uptake and retention, which might cause expansion of leaf and root tissues of transgenic tobacco (Clément et al., 2006, 2007). Expressions of *rolC* and tobacco endogenous *rolC* homolog also induce similar effects on sucrose uptake, suggesting a functional relationship between *rolC* and* 6b* (Mohajjel-Shoja et al., 2011).

In several *Arabidopsis* mutants that have defects in the establishment of leaf adaxial–abaxial polarity, similar leaf morphological defects are observed (McConnell and Barton, 1998; Eshed et al., 2001; Ueno et al., 2007; Kojima et al., 2011; Ishibashi et al., 2012). A model has been proposed whereby the appropriate cell differentiation of leaf adaxial and abaxial domains is required for the development of flat leaf lamina, and thus defects in the establishment of leaf adaxial–abaxial polarity cause inhibition of the leaf laminar expansion (Waites and Hudson, 1995; Emery et al., 2003). In the transgenic tobacco plants that express *6b*, the adaxial–abaxial polarity of leaves might be affected as a consequence of aberrant expression of tobacco genes that are involved in the establishment of such a polarity.

### PHENOTYPES OF *Arabidopsis* PLANTS THAT EXPRESS *6b*

In the transgenic *Arabidopsis* plants that express the *6b* gene, morphological defects were also observed, such as the upwardly curled leaves, protrusion at the leaf abaxial side, and apparent serrations in the leaf lamina (**Figure 1C**; Terakura et al., 2006). In addition, the *Arabidopsis* transgenic plants that express *6b* genes from the *Agrobacterium vitis* AB4 strain or *A. tumefaciens* Tm4 strain generate rod-like shaped leaves with short lamina along the longitudinal axis and a long petiole (Helfer et al., 2003). These phenotypes are somewhat similar to those of the *Arabidopsis* plants in which the *ASYMMETRIC LEAVES2* (*AS2*) gene is ectopically overexpressed (Iwakawa et al., 2002).

To understand the molecular bases for the above mentioned phenotypes of *Arabidopsis* transgenic plants, Terakura et al. (2006) measured the transcript levels of a number of genes related to leaf morphology and cell division. The results showed increases in levels of transcripts of the *Cyclin B1* and* AtNACK1* genes (cell cycle markers); class 1 *KNOX* genes (markers for meristem maintenance: *STM, BP, KNAT2,* and *KNAT6*); and *CUC* genes (markers for meristem maintenance and separation of organs from the meristem: *CUC1, CUC2,* and *CUC3*). These data suggest that the meristematic state and cell division competence are increased in *6b*-transgenic *Arabidopsis* plants. Transcript levels of *HD-ZIP III* and *ASYMMETRIC LEAVES1* (markers for adaxial development) or of *KAN1* and *FIL* (markers for abaxial development), however, were not significantly affected by *6b* expression (Terakura et al., 2006). The results of microarray analyses do not allow us to simply explain the molecular basis of the abnormal leaf morphology of the *6b*-transgenic *Arabidopsis*.

### THE *6b* GENE ENHANCES DEVELOPMENTALLY INDETERMINATE STATES AND CELL PROLIFERATION POTENTIAL IN PLANTS

The tobacco leaf disks that are transcribed under control of the *Cauliflower mosaic virus* 35S promoter generate white or green abnormal cell proliferation. During the callus formation by *6b* expression, the amounts of endogenous phytohormones, cytokinin and auxin, are not changed (Wabiko and Minemura, 1996). It seems likely that the *6b* gene might directly or indirectly induce the expression of genes required for formation of calli. Gene *6b* also suppresses both the shoot formation induced by cytokinins *in vitro* (Spanier et al., 1989; Wabiko and Minemura, 1996) and the root formation induced by auxin and *rolABC* (Tinland et al., 1990), suggesting that 6b protein not only induces the cell proliferation under hormone-free conditions but also suppresses the cell differentiation induced by cytokinins and auxins.

Transcripts levels of genes for meristem (stem cell) maintenance, class 1 *KNOX* genes, are increased in *Arabidopsis* and that the expression of class 1 *KNOX* genes of tobacco (*NTH15, NTH1, NTH20*, and *NTH22*) is also increased in the leaves of *6b*-transgenic tobacco plants (Terakura et al., 2006). These results suggest an increase in the undifferentiated states of cells in these *6b*-transgenic plants.

## MOLECULES INTERACTING WITH 6b PROTEIN

To unveil the molecular functions of 6b protein, Kitakura et al. (2002, 2008) and Terakura et al. (2007) have sought for 6b-interacting proteins of the tobacco cultured cell line BY-2. They screened tobacco cDNA libraries by yeast two-hybrid systems and eventually identified three positive cDNAs of interest in terms of tumorigenicity, cell division competency, and the formation of morphologically malformed leaves. Kitakura et al. (2002, 2008) identified two of these genes designated *Nicotiana* Six-b Interacting Protein 1 and 2 (*NtSIP1* and *NtSIP2*); and Terakura et al. (2007) identified a third gene, *NtSIP3*.

### NtSIP1 IS LOCALIZED TO NUCLEI AND ENHANCES THE NUCLEAR LOCALIZATION OF THE 6b PROTEIN IN TOBACCO CELLS

*NtSIP1* encodes a protein that consists of 318 amino acid residues with a molecular mass of 34.8 kD (Kitakura et al., 2002). The region from residue 72 to residue 131 of NtSIP1 is predicted to form three-helices with short intervening loops. This region is similar to the triple helix motif of rice transcription factor GT-2, which controls the transcription level of the *PHYA* gene (Dehesh et al., 1992). There are two basic regions that resemble a NLS. In the *Arabidopsis* genome, several homologs of the *NtSIP1* gene exist. The amino acid residues of NtSIP1 are 43% identical to those of the predicted protein of F14P22.220, 36% identical to those of MOP10_9, 34% identical to those of F9F8.9, and 27% identical to those of F11B9.6. Phylogenetic tree analysis indicates that NtSIP1 is closely related to F14P22.220 of *Arabidopsis*. Amino acid sequences corresponding to a triple helix motif and NLSs are conserved in all of these deduced sequences. In BY-2 cells, NtSIP1 localizes in the nuclei. Accumulated NtSIP1 transcripts are detected in roots, stems, mature leaves, and shoot apices that contain the shoot apical meristem, and those transcript levels are significantly higher in shoot apices than in other organs (Kitakura et al., 2002). NtSIP1 enhances the nuclear localization of 6b protein in BY-2 cells, and the acidic region of 6b is necessary for the interaction with NtSIP1 and the enhancement of the nuclear localization of 6b (Kitakura et al., 2002).

### NtSIP2 HAS HOMOLOGY TO TNP1 ENCODED BY THE TRANSPOSABLE ELEMENT Tam1 OF *Antirrhinum majus*

The *NtSIP2* gene is predicted to encode a protein with 424 amino acid residues, and the predicted protein exhibits homology to TNP1 encoded by the transposable element Tam1 of *A. majus* (Kitakura et al., 2008). The predicted amino acid sequence of NtSIP2 is 30% identical and 50% similar to that of the TNP1 protein (Nacken et al., 1991). NtSIP2 has three short stretches of basic amino acid residues that are similar to the NLS sequence. The *NtSIP2* transcripts are detected in a variety of plant tissues without significant quantitative difference. Almost all NtSIP2 protein is localized to the nucleolus. Since significant amounts of the 6b protein are also detected in the nucleolus, NtSIP2 in the nucleolus might be interacted with the 6b protein. NtSIP2 might play a role in transporting 6b into the nucleolus by binding activity and the 6b-NtSIP2 complex might act as a transcriptional repressor within the nucleolus.

### NtSIP3

#### *NtSIP3* encodes histone H3

By using the yeast two-hybrid system with a *6b* sequence lacking the DNA region corresponding to the acidic region (**Figure 1A**), Terakura et al. (2007) identified two tobacco cDNAs for members of the tobacco histone H3 protein family. The predicted amino acid sequences of the two tobacco clones are identical to those of histone H3.1 (DNA replication-dependent H3) and H3.2 (replication-independent H3) of *Arabidopsis* (Johnson et al., 2004), respectively, which are collectively designated as NtSIP3. The tobacco histone H3.2 clone was used for* in vitro* binding assay and* in vivo* binding by BiFC (bimolecular fluorescence complementation) analysis. The results of these experiments have shown that the 6b protein directly interacts with tobacco histone H3.2* in vitro* and *in vivo*. Protein 6b also binds to *Arabidopsis* histone H3.1 (At3g27360.1) and H3.2 (At4g40030.1) *in vitro*. In the tobacco BY-2 cells, 6b protein interacts with chromatin. The amino acid sequence of 6b without the acidic region binds to the histone fold region of histone H3.2 (60–135 residues) and the C-terminal region of 6b (185–208 residues) is required for the binding (Terakura et al., 2007).

**FIGURE 2:**
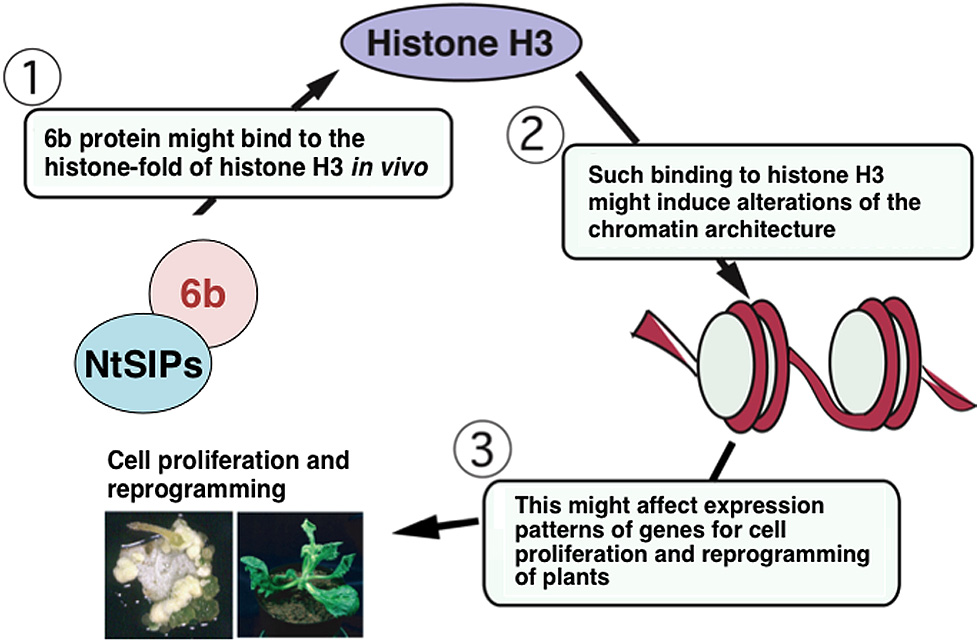
**Working hypothesis of 6b action.** See details in text. NtSIPs represent NtSIP1 or NtSIP2. They might independently bind to 6b and/or both might simultaneously bind to 6b.

It was examined whether 6b can bind other core histones (H2A, H2B, and H4). The results show that it binds to histone H3 specifically (Terakura et al., 2007). Since the histone fold consists of three α helices that are involved in the formation of nucleosomes, 6b protein might have some effect on the formation of the nucleosome* in vivo* (**Figure 2**).

#### 6b protein has histone chaperone activity

Histone chaperones are known to be factors that enhance the formation of nucleosomes* in vitro* (Loyola and Almouzni, 2004). Histone chaperones, such as HIRA (histone regulator A) and ASF1 (antisilencing factor 1), bind to the histone fold domains of core histones (Munakata et al., 2000; Ray-Gallet et al., 2002). Furthermore, histone chaperones, such as NAP-1 (nucleosome assembly protein-1; Ishimi and Kikuchi, 1991), nucleophosmin/B23 (Okuwaki et al., 2001), yeast FK506bp (Kuzuhara and Horikoshi, 2004), and nucleolin (Angelov et al., 2006), contain acidic regions of amino acid sequence. The β sheet structure of ASF1 is required for the interaction with histone, and other histone chaperones, such as NAP-1, RbAp46/48, p60 (a component of Chromatin Assemby Factor-1 complex) and HIRA, are also predicted to contain similar β sheet structures (English et al., 2006). Intriguingly, the C-terminal amino acids that are required for the interaction with histone H3 are predicted to form the β-sheet structure. The 6b protein is similar to known histone chaperones in terms of the following three points: (1) the physical association of 6b with the histone fold of histone H3; (2) the presence of the β-sheets in the C-terminus of 6b (Wang et al., 2011); and (3) the presence of the acidic region in 6b.

By using a supercoiling assay, 6b protein was demonstrated to have histone chaperone-like activity* in vitro* (Terakura et al., 2007). The C-terminal region is required for the cell division-stimulating activity, interaction with histone H3, and histone chaperon-like activity of 6b, suggesting that such activity is related to the ability of 6b to induce cell proliferation. AB6b from *A. vitis* AB4 strain also binds to histone H3 and has histone chaperone-like activity. Similarly as for *AK6b, AB6b* also induces severe morphological defects in transfected plants (Helfer et al., 2003). Histone chaperones affect the histone dynamics, such as histone conservation (Dutta et al., 2001), histone transport (Ito et al., 1996; Mosammaparast et al., 2002), and chromatin structure conversion (Smith and Stillman, 1989; Belotserkovskaya et al., 2003; Adkins et al., 2004; Boeger et al., 2004; Schermer et al., 2005) to control the transcription and DNA replication in the nucleus (Loyola and Almouzni, 2004; Polo and Almouzni, 2006). The 6b protein might act as a histone chaperone* in vivo* and regulate the chromatin structure to affect gene transcription for cell proliferation and plant differentiation (**Figure 2**).

## FUTURE PROSPECTS

Through the analyses of its interacting molecules, *Agrobacterium* 6b protein functions in the nucleus and the nucleolus of plant cells, in which it might interact with NtSIP1, NtSIP2, and histone H3 and act as a histone chaperone to alter expression patterns of genes related to tumorigenicity, cell proliferation, and reprogramming of plant cells (**Figure 2**). It is generally accepted that histone chaperons affect epigenetic status of chromatin mainly by replacing canonical histones with replacement histones (differing in several amino acid residues from canonical ones), which can alter patterns of gene expression (Skene and Henikoff, 2013). Formation of crown gall tumors largely depends on expression of genes for enzymes involved in cytokinin and auxin biosynthesis. The histone chaperone activity of 6b might affect chromatin structures to regulate the expression levels of the genes related to auxin and cytokinin biosynthesis. Plants have various silencing mechanisms to repress expression of transgenes, such as those from pathogens, by chromatin remodeling, DNA methylation, histone modification, and/or the RNAi system (Zilberman and Henikoff, 2005). Expression of the phytohormone biosynthesis genes in the T-DNA region might be down-regulated by the silencing mechanisms of host plants. Histone chaperone activity of 6b might compromise actions of the plant silencing systems. Suppression of the gene silencing systems by *6b* might promote expression of genes for auxin and cytokinin biosynthesis, which might affect the host range of *Agrobacterium* strains to induce crown gall tumors (Hooykaas et al., 1988). Genetic manipulation of the *6b* gene might contribute to improving *Agrobacterium*-mediated gene transfer technologies.

Apart from the histone chaperone activity of 6b, the crystal structure of this protein exhibits a certain extent of the structural similarity to an ADP ribosylation factor of *Arabidopsis*, and the 6b protein bears a biochemical role in ADP ribosylation under conditions used by Wang et al. (2011). Relationships between this activity, phenotypes and alteration of patterns of gene expression generated by 6b remain to be elucidated.

It is notable that transcript levels of cell division- and meristem-related genes such as class 1 *KNOX* and *CYCLIN* genes are increased. These data support the hypothesis that the cell proliferation potential and meristematic states might be enhanced by *6b*. Accumulation levels of transcripts of a number of auxin-inducible genes, such as those of the *GH3*, *IAA,* and *ACS* family genes, are decreased in *6b-*expressing transgenic *Arabidopsis* plants (Terakura et al., 2007). These results might imply that various physiological and cellular statuses that are controlled by such auxin-inducible genes might be altered by *6b* expression. It should be critical to identify direct target genes of 6b to uncover molecular frameworks of cell reprogramming and intriguing to examine whether plant endogenous histone chaperons might have reprogramming activities.

## AUTHOR CONTRIBUTIONS

Nanako Ishibashi and Yasunori Machida designed the outline of this article. Nanako Ishibashi wrote Abstract, Introduction, Section “Characteristics of Transgenic Plants that Express 6b,” and References. Saeko Kitakura wrote the Section “NtSIP1 is Localized to Nuclei and Enhances the Nuclear Localization of the 6b Protein in Tobacco Cells.” Chiyoko Machida wrote the Sections “Structural Feature of 6b Protein” and “NtSIP2 has Homology to TNP1 Encoded by the Transposable Element Tam1 of *Antirrhinum majus,*” and contributed to revise the original title by the addition of “Reprogramming” to the present form. Chiyoko Machida wrote the Section “Future Prospects” to revise the original version. Shinji Terakura and Yasunori Machida wrote the Section “NtSIP3.” Yasunori Machida wrote the original part of the Section “Future prospects,” and review and edit the entire manuscript. All authors are accountable for all aspects of the description of this article in ensuring that questions related to the accuracy or integrity of any part of the article are appropriately investigated and resolved.

## Conflict of Interest Statement

The authors declare that the research was conducted in the absence of any commercial or financial relationships that could be construed as a potential conflict of interest.
